# Multi-EPL: Accurate multi-source domain adaptation

**DOI:** 10.1371/journal.pone.0255754

**Published:** 2021-08-05

**Authors:** Seongmin Lee, Hyunsik Jeon, U. Kang

**Affiliations:** Seoul National University, Seoul, Republic of Korea; Fuzhou University, CHINA

## Abstract

*Given multiple source datasets with labels, how can we train a target model with no labeled data?* Multi-source domain adaptation (MSDA) aims to train a model using multiple source datasets different from a target dataset in the absence of target data labels. MSDA is a crucial problem applicable to many practical cases where labels for the target data are unavailable due to privacy issues. Existing MSDA frameworks are limited since they align data without considering labels of the features of each domain. They also do not fully utilize the target data without labels and rely on limited feature extraction with a single extractor. In this paper, we propose Multi-EPL, a novel method for MSDA. Multi-EPL exploits label-wise moment matching to align the conditional distributions of the features for the labels, uses pseudolabels for the unavailable target labels, and introduces an ensemble of multiple feature extractors for accurate domain adaptation. Extensive experiments show that Multi-EPL provides the state-of-the-art performance for MSDA tasks in both image domains and text domains, improving the accuracy by up to 13.20%.

## Introduction

*Given multiple source datasets with labels, how can we train a target model with no labeled data?* Large training data are essential for training deep neural networks. Collecting abundant data is, unfortunately, an obstacle in practice; even if enough data are obtained, manually labeling those data is prohibitively expensive. Using other available or much cheaper datasets would be a solution for these limitations; however, indiscriminate usage of other datasets often brings severe generalization error due to the presence of dataset shifts [1]. Unsupervised domain adaptation (UDA) tackles these problems where no labeled data from the target domain are available, but labeled data from other source domains are provided. Finding out domain-invariant features has been the focus of UDA since it allows knowledge transfer from the labeled source dataset to the unlabeled target dataset. There have been many efforts to transfer the knowledge from a single source domain to a target one. Most recent frameworks minimize the distance between two domains by deep neural networks and distance-based techniques such as discrepancy regularizers [2–4], adversarial networks [5, 6], and generative networks [7–9].

**Fig 1:**
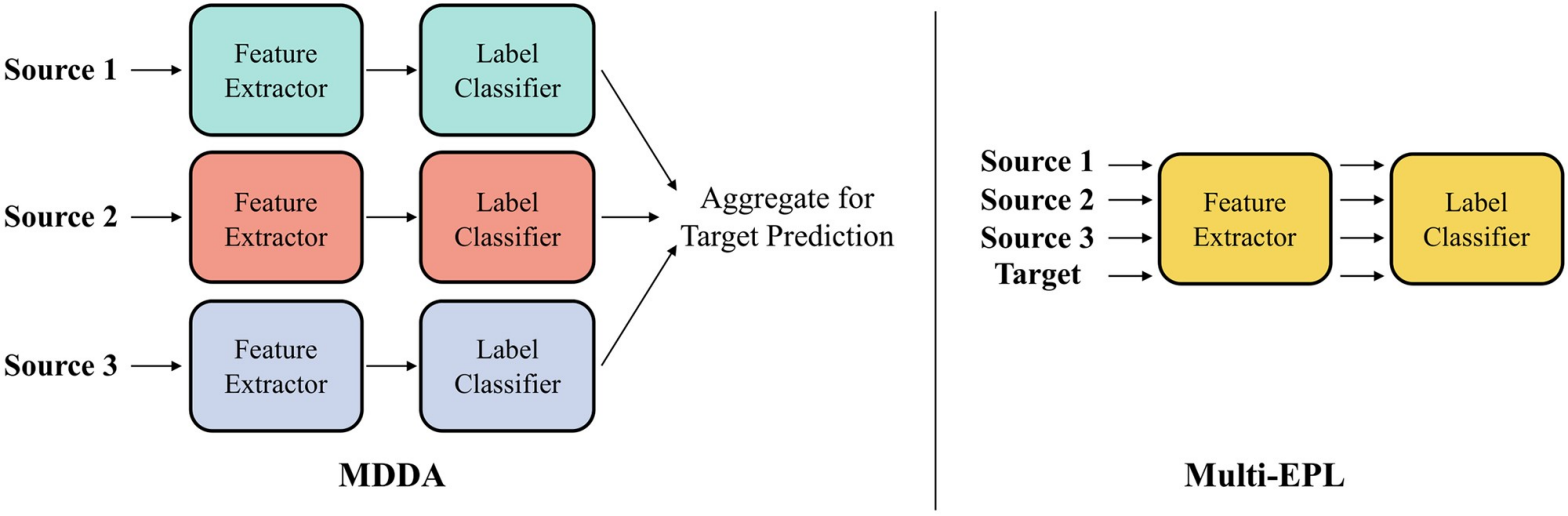
Overall model structure of MDDA and Multi-EPL. To handle 3 source domains, most existing methods deploy 3 different sets of deep neural networks, while one single set is enough for Multi-EPL. This allows Multi-EPL to use ensemble learning without an excessive cost of model complexity.

While the above-mentioned approaches consider a single source, we address multi-source domain adaptation (MSDA), which is very crucial and more practical in real-world applications as well as more challenging. MSDA is able to bring significant performance enhancement by virtue of accessibility to multiple datasets as long as multiple domain shift problems are resolved. Previous works have extensively presented both theoretical analysis [10–15] and models [14, 16–20] for MSDA. MDAN [14], DCTN [16], and MDDA [18] build adversarial networks for each source domain to generate features domain-invariant enough to confound domain classifiers. However, these approaches do not encompass the interactions among source domains, counting only shifts between source and target domain. M^3^SDA [17] adopts a moment matching strategy but makes the unrealistic assumption that matching the marginal probability *p*(**x**) would guarantee the alignment of the conditional probability *p*(**x**|*y*). Most of these methods also do not fully exploit the knowledge of the target domain, imputing to the inaccessibility of the labels. Furthermore, these methods require individual deep neural networks for each source domain as described in Fig 1, which have great redundancy and significantly increase the overall model complexity. LtC-MSDA configures prototypes of the features from each domain and learns the interaction between multiple domains deploying GCN. However, summarizing each domain into only one prototype cannot fully represent the feature distributions of the domain and therefore deteriorates the performance.

**Fig 2:**
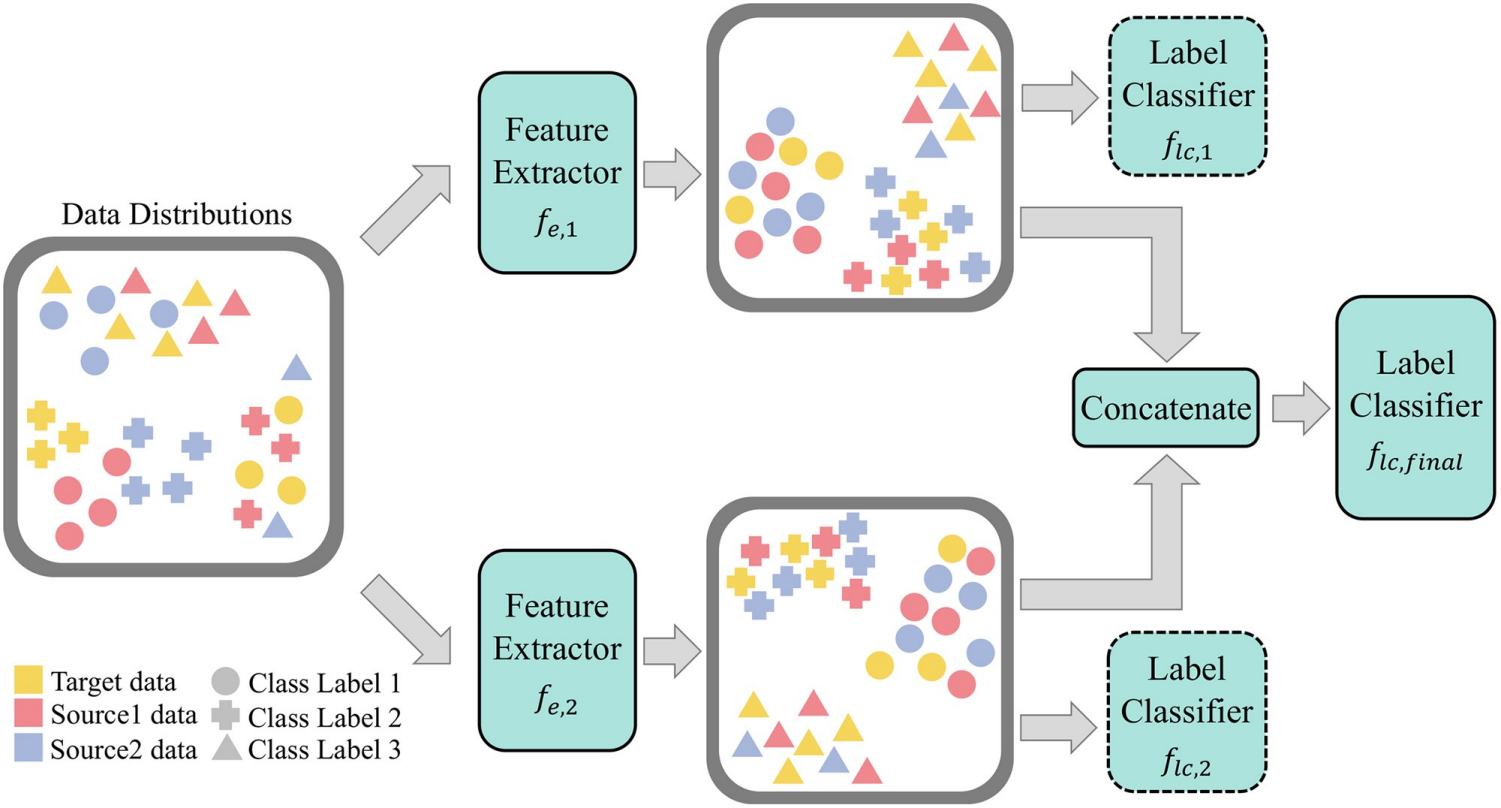
Illustration of Multi-EPL. Multi-EPL consists of 2 pairs of feature extractors and label classifiers, and one final label classifier. Colors and symbols of the markers indicate domains and class labels of the data, respectively. The networks with the solid line are used for inference while the ones with the dashed line are used only for training.

**Fig 3:**
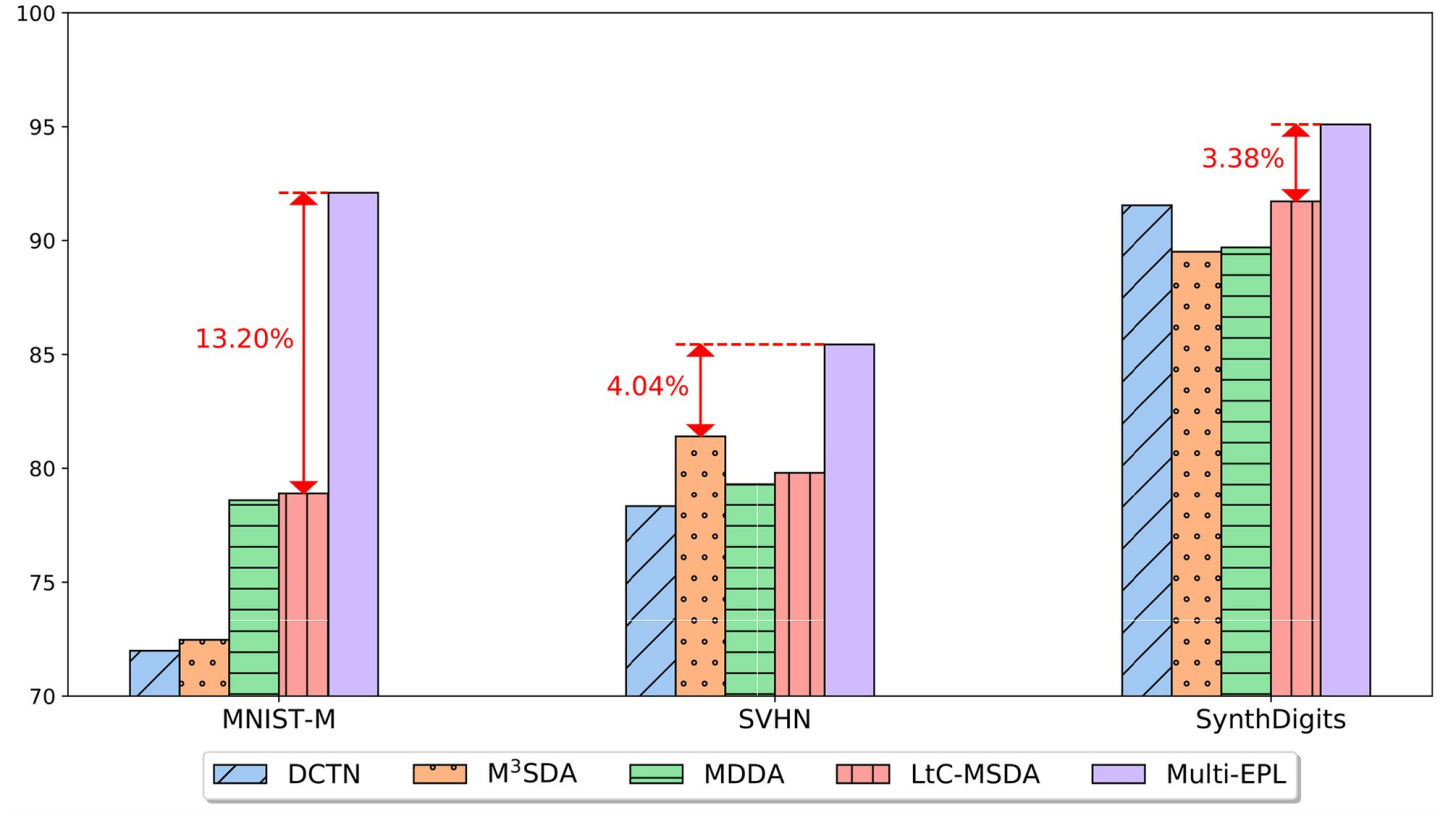
Accuracy of Multi-EPL and its competitors on 3 cases with Digits-Five datasets.

In this paper, we propose Multi-EPL (Multi-source domain adaptation with Ensemble of feature extractors, Pseudolabels, and Label-wise moment matching), a novel MSDA framework that mitigates the limitations of these methods of not explicitly considering conditional probability *p*(**x**|*y*), and having great redundancy in their models. Multi-EPL is illustrated in Fig 2. Multi-EPL aligns the conditional probability *p*(**x**|*y*) by utilizing label-wise moment matching. We employ pseudolabels for the inaccessible target labels to maximize the usage of the target data. Moreover, we generate an ensemble of features from multiple feature extractors to capture rich information about labels. Extensive experiments show the superiority of Multi-EPL (see Fig 3).

Our contributions are summarized as follows:

- **Method**. We propose Multi-EPL, a novel approach for MSDA that effectively and efficiently obtains domain-invariant features from multiple domains by matching conditional probability *p*(**x**|*y*), utilizing pseudolabels for inaccessible target labels to fully exploit target data, handling all the source domains with one single neural network, and using an ensemble of multiple feature extractors for further enhancement. It allows domain-invariant features to be extracted, capturing the intrinsic differences of labels.
- **Experiments**. We conduct extensive experiments on image and text datasets. We show that 1) Multi-EPL provides the state-of-the-art performance, and 2) each of our main ideas significantly contributes to the superior performance.

In the rest of this paper, we first introduce the related works and describe our proposed method. Then, we experimentally evaluate the performance of Multi-EPL and its competitors. The code for Multi-EPL can be found in https://github.com/snudatalab/MultiEPL. Frequently used symbols are summarized in Table 1.

**Table 1:** Table of symbols.

Symbols	Definition
*N*	Number of source domains.
X	Data space.
C	Label set.
$\mu_{D}$	Data distribution of the domain D.
$l_{D}$	Labeling function of the domain D.
$S_{i}=\left(\mu_{S_{i}},l_{S_{i}}\right)$	*i*-th source domain.
$T=(\mu_{T},l_{T})$	Target domain.
$n_{S_{i}}$	Number of instance in *i*-th source dataset.
$n_{T}$	Number of instance in the target dataset.
$X_{S_{i}}={\left\{\left(x_{j}^{S_{i}},y_{j}^{S_{i}}\right)\right\}}_{j=1}^{n_{S_{i}}}$	*i*-th source dataset. $x_{j}^{S_{i}}$: *j*-th instance in $X_{S_{i}}$, $y_{j}^{S_{i}}$: label of $x_{j}^{S_{i}}$.
$X_{T}={\left\{x_{j}^{T}\right\}}_{j=1}^{n_{T}}$	Target dataset. $x_{j}^{T}$: *j*-th instance in $X_{T}$.

## Related works

### Single-source domain adaptation

Given a labeled source dataset and an unlabeled target dataset, single-source domain adaptation aims to train a model that performs well on the target domain. The challenge of single-source domain adaptation is to reduce the discrepancy between the two domains and to obtain appropriate domain-invariant features. Various discrepancy measures such as Maximum Mean Discrepancy (MMD) [2–4, 21, 22] and KL divergence [23] have been used as regularizers. Inspired by the insight that the domain-invariant features should exclude the clues about its domain, constructing adversarial networks against domain classifiers has shown superior performance. [7] and [9] deploy GAN to transform data across the source and target domains, while [5] and [6] leverage the adversarial networks to extract common features of the two domains. Unlike these works, we focus on *multiple* source domains.

### Multi-source domain adaptation

Single-source domain adaptation should not be naively employed for multiple source domains due to domain shifts. Many previous works have tackled Multi-source Domain Adaptation (MSDA) problems theoretically. [11] establishes distribution weighted combining rule that the weighted combination of source hypotheses is a good approximation for the target hypothesis. The rule is further extended to a stochastic case with joint distribution over the input and the output space in [13]. [12] proposes a general theory of how to sift appropriate samples out of multi-source data using expected loss. Efforts to find out transferable knowledge from multiple sources from the causal viewpoint are made in [24]. There have been salient studies on the learning bounds for MSDA. [10] finds the generalization bounds based on HΔH-divergence, which are further tightened by [14].

Frameworks for MSDA have been presented as well. [14] proposes learning algorithms based on the generalization bounds for MSDA. DCTN [16] resolves domain and category shifts between source and target domains via adversarial networks. TMDA [25] aligns multiple domains utilizing clustering and adversarial training. M^3^SDA [17] associates all the domains into a common distribution by matching the moments of the feature distributions of multiple domains. In [26], attempts to find out the common latent space of source and target domains are made, focusing on the visual sentiment classification tasks. MDDA [18] employs Wasserstein distance to figure out which data from which source domains are closely related to the target data. In LtC-MSDA [19], the interactions among multiple domains are learned by constructing a knowledge graph. However, most of these methods do not consider multimode structures [27] that differently labeled data follow distinct distributions, even if they are drawn from the same domain. Also, the domain-invariant features in these methods contain the label information for only one label classifier which leads these methods to miss a large amount of label information. Differently from these methods, our framework fully considers the multimode structures, handles the data distributions in a label-wise manner, and minimizes the label information loss considering multiple label classifiers.

### Moment matching

Moment matching strategy has been used to minimize the discrepancy between source and target domains in domain adaptation. MMD regularizer [2–4, 21, 22] can be interpreted as the first-order moment matching while [28] addresses second-order moment maching of source and target distributions. [29] investigates the effect of higher-order moment matching. M^3^SDA [17] demonstrates that moment matching yields remarkable performance also with multiple sources. While previous works have focused on matching the moments of marginal distributions for single-source adaptation, we handle conditional distributions in multi-source scenarios.

## Methods

In this section, we describe our proposed method, Multi-EPL. We first formulate the problem definition and describe our main ideas. Then, we elaborate on how to match label-wise moment with pseudolabels and extend the approach by adding the concept of ensemble learning. Fig 2 shows the overview of Multi-EPL.

### Problem definition

Given a set of labeled datasets from *N* source domains $S_{1},\ldots ,S_{N}$ and an unlabeled dataset from a target domain T, we aim to construct a model that minimizes the test error on T. We formulate source domain $S_{i}$ as a tuple of the data distribution $\mu_{S_{i}}$ on data space X and the labeling function $l_{S_{i}}:S_{i}=\left(\mu_{S_{i}},l_{S_{i}}\right)$. Source dataset drawn with the distribution $\mu_{S_{i}}$ is denoted as $X_{S_{i}}={\left\{\left(x_{j}^{S_{i}},y_{j}^{S_{i}}\right)\right\}}_{j=1}^{n_{S_{i}}}$, where $n_{S_{i}}$ is the number of instance in $X_{S_{i}}$. Likewise, the target domain and the target dataset are denoted as $T=(\mu_{T},l_{T})$ and $X_{T}={\left\{x_{j}^{T}\right\}}_{j=1}^{n_{T}}$, respectively, where $n_{T}$ is the number of instance in $X_{T}$. We narrow our focus down to homogeneous settings in classification tasks: all domains share the same data space X and label set C.

### Overview

We propose Multi-EPL based on the following observations: 1) existing methods focus on aligning the marginal distributions *p*(**x**) not the conditional ones *p*(**x**|*y*), 2) knowledge of the target data is not fully employed as no target label is given, 3) existing methods that require separate neural networks for each source domain have considerable inefficiency in model size, and 4) there is a large amount of loss in label information since domain-invariant features are extracted for only one label classifier. Designing a method to solve these limitations entails the following challenges:

1. **Matching conditional distributions**. How can we align the conditional distribution, *p*(**x**|*y*), of multiple domains, not the marginal one, *p*(**x**)?
2. **Exploitation of the target data**. How can we fully exploit the knowledge of the target data despite the absence of the target labels?
3. **Maximization of the model efficiency**. How can we maximize the model efficiency and performance?

We propose the following main ideas to address the challenges:

1. **Label-wise moment matching**. We match the *label-wise* moments of the domain-invariant features so that the features with the same labels have similar distributions regardless of their original domains. This improves not only adaptation but also classification performance compared to the previous methods, which align features not considering labels and therefore cannot clearly separate differently labeled instances.
2. **Pseudolabels**. We use pseudolabels as alternatives to the target labels. While the existing MSDA methods have made only limited use of target data, this allows the intrinsic properties related to the label prediction of each target instance to be better reflected.
3. **Ensemble of feature representations**. We integrate multiple neural networks, each of which handles each source domain, into one neural network. For further improvement, we propose a variant of ensemble learning to concatenate features from multiple feature extractors. This enhances model performance without an extreme increase in model size, whereas the existing methods have significantly increased model size for better performance.

Our model Multi-EPL consists of two pairs of feature extractor and label classifier, (*f*_*e*,1_, *f*_*lc*,1_) and (*f*_*e*,2_, *f*_*lc*,2_), and one final label classifier, *f*_*lc*,*final*_ as shown in Fig 2. The feature extractors distill the domain-invariant features, which are aligned to have similar distributions regardless of their domains. Then, the label classifiers take the features from the corresponding feature extractor as inputs and predict their labels. Meanwhile, the features from *f*_*e*,1_ and *f*_*e*,2_ are concatenated and fed into the final label classifier *f*_*lc*,*final*_. The label prediction of *f*_*lc*,*final*_ is used for the final inference.

### Label-wise moment matching with pseudolabels

We describe how Multi-EPL matches conditional distributions *p*(**x**|*y*) of the features from multiple distinct domains. In Multi-EPL, a feature extractor *f*_*e*_ and a label classifier *f*_*lc*_ lead the features to be domain-invariant and label-informative at the same time. The feature extractor *f*_*e*_ extracts features from data, and the label classifier *f*_*lc*_ receives the features and predicts the labels for the data. We train *f*_*e*_ and *f*_*lc*_, according to the losses for *label-wise moment matching* and *label classification*, which make the features domain-invariant and label-informative, respectively.

#### Label-wise moment matching

To achieve the alignment of domain-invariant features, we define a label-wise moment matching loss as follows:

$$\begin{array}{c} L_{lmm,K}=\frac{1}{\left|C\right|}\frac{1}{N}\sum_{k=1}^{K}\sum_{i=1}^{N}\sum_{c\in C}{∥\frac{1}{n_{S_{i},c}}\sum_{j;y_{j}^{S_{i}}=c}f_{e}{\left(x_{j}^{S_{i}}\right)}^{k}-\frac{1}{n_{T,c}}\sum_{j;y_{j}^{T}=c}f_{e}{\left(x_{j}^{T}\right)}^{k}∥}_{2}, \end{array}$$ (1)

where *K* is a hyperparameter indicating the maximum order of moments considered by the loss, and $n_{D,c}$ is the number of data labeled as *c* in $X_{D}$. We introduce *pseudolabels* to determine the label *c* for the target data, which are determined by the outputs of the model currently being trained, to manage the absence of the ground truths for the target data. In other words, we compute $f_{lc}\left(f_{e}\left(x^{T}\right)\right)$ using *f*_*lc*_ and *f*_*e*_ trained up to the previous iteration step to give the pseudolabels to the target data $x^{T}$.

The L2 norm term in Eq 1 measures how much *k*-th order moments of the features labeled as *c* are different when it comes to the source domain $S_{i}$ and the target domain T. The sum of the term for every possible *c*, *i*, and *k* gives the discrepancy of the feature distributions between the source domains and the target domain. By minimizing $L_{lmm,K}$, the feature extractor *f*_*e*_ aligns data from multiple domains by bringing consistency in distributions of the features with the same labels. The data with distinct labels are aligned independently, taking account of the multimode structures that differently labeled data follow different distributions.

#### Label classification

The label classifier *f*_*lc*_ gets the features projected by *f*_*e*_ as inputs and makes the label predictions. The *label classification loss* is defined as follows:

$$\begin{array}{c} L_{lc}=\frac{1}{N}\sum_{i=1}^{N}\frac{1}{n_{S_{i}}}\sum_{j=1}^{n_{S_{i}}}L_{ce}\left(f_{lc}\left(f_{e}\left(x_{j}^{S_{i}}\right)\right),y_{j}^{S_{i}}\right), \end{array}$$ (2)

where $L_{ce}$ is the softmax cross-entropy loss. Minimizing $L_{lc}$ separates the features with different labels so that each of them becomes label-distinguishable.

### Ensemble of feature representations

In this section, we introduce ensemble learning for further enhancement. Features extracted with the method described in the previous section contain the label information for a single label classifier. However, each label classifier leverages only limited label characteristics, and thus the conventional scheme to adopt only one pair of feature extractor and label classifier captures only a small part of the label information. Our idea is to leverage an ensemble of multiple pairs of feature extractors and label classifiers in order to make the features to be more label-informative.

We train two pairs of feature extractor and label classifier in parallel following the label-wise moment matching approach explained in the previous section. We denote the two (feature extractor, label classifier) pairs as (*f*_*e*,1_, *f*_*lc*,1_) and (*f*_*e*,2_, *f*_*lc*,2_), and the resultant features from each feature extractor as *feat*_1_ and *feat*_2_ respectively. After obtaining two different feature mappings, we concatenate the two into one vector *feat*_*final*_ = *concat*(*feat*_1_, *feat*_2_). The final label classifier *f*_*lc*,*final*_ takes the concatenated feature as input and predicts the label of the feature.

### Multi-EPL: Accurate multi-source domain adaptation

Our final model Multi-EPL consists of two pairs of feature extractor and label classifier, (*f*_*e*,1_, *f*_*lc*,1_) and (*f*_*e*,2_, *f*_*lc*,2_), and one final label classifier, *f*_*lc*,*final*_. We train the model in an iterative manner where each iteration is composed of two steps. We first train the entire model except for the final label classifier with the loss L:

$$\begin{array}{c} L=\sum_{n=1}^{2}\left(L_{lc,n}+\alpha L_{lmm,K,n}\right), \end{array}$$ (3)

where $L_{lc,n}$ is the label classification loss of the classifier *f*_*lc*,*n*_, $L_{lmm,K,n}$ is the label-wise moment matching loss of the feature extractor *f*_*e*,*n*_, *α* is a hyperparameter that weights each of the loss term, and *K* is the hyperparameter for the maximum order of moments in $L_{lmm,K,n}$. Then, the final label classifier is trained with respect to the label classification loss $L_{lc,final}$ using the concatenated features from the multiple feature extractors. We repeat these two steps over and over until the number of iterations reaches the predetermined number of epochs.

## Experimental results

We conduct experiments to answer the following questions.

**Q1 Accuracy**. How well does Multi-EPL perform in classification tasks?
**Q2 Ablation Study**. How much does each component of Multi-EPL contribute to performance improvement?
**Q3 Effects of Degree of Ensemble**. How does the performance change as the number of the pairs of the feature extractor and the label classifier increases?
**Q4 Parameter Efficiency**. What is the parameter efficiency of Multi-EPL compared to the other methods?

### Experimental settings

#### Datasets

We use three collections of datasets, Digits-Five, Office-Caltech10 [30], and Amazon Reviews [31], listed in Table 2. Digits-Five consists of five datasets for digit recognition: MNIST [32], MNIST-M [33], SVHN [34], SynthDigits [33], and USPS [35]. We set one of them as a target domain and the rest as source domains. Following the conventions in prior works [16, 17], we randomly sample 25000 instances from the source training set and 9000 instances from the target training set to train the model except for USPS for which the whole training set is used. The entire test set is exploited to evaluate the performance. Office-Caltech10 is for image classification with 10 categories that Office31 dataset and Caltech dataset have in common. It involves four different domains: Amazon, Caltech, DSLR, and Webcam. We double the number of instances by data augmentation and exploit all the original instances and augmented instances as training and test sets, respectively. Amazon Reviews contains customers’ reviews on 4 product categories: Books, DVDs, Electronics, and Kitchen appliances. The instances are encoded into 5000-dimensional vectors and are labeled as being either positive or negative depending on their sentiments. We set each of the four categories as a target and the rest as sources. For all the domains, 2000 instances are sampled for training, and the rest of the data are used for the test.

**Table 2:** Summary of datasets.

	Datasets	Features	Class#	Training	Test
**Digits-Five**	MNIST	1x28x28	10	60000	10000
	MNIST-M	3x32x32	10	59001	9001
	SVHN	3x32x32	10	73257	26032
	SynthDigits	3x32x32	10	479400	9553
	USPS	1x16x16	10	7291	2007
**Office-Caltech10**	Amazon	3x300x300	10	958	958
	Caltech	Variable	10	1123	1123
	DSLR	3x1000x1000	10	157	157
	Webcam	Variable	10	295	295
**Amazon Reviews**	Books	5000	2	2000	4465
	DVDs	5000	2	2000	3586
	Electronics	5000	2	2000	5681
	Kitchen appliances	5000	2	2000	5945

#### Competitors

We use 5 MSDA algorithms, DCTN [16], M^3^SDA, M^3^SDA-*β* [17], MDDA [18], and LtC-MSDA [19] with state-of-the-art performances as baselines. All the frameworks share the same architecture for the feature extractor and the label classifier for consistency. For Digits-Five, we use convolutional neural networks based on LeNet5 [32]. For Office-Caltech10, ResNet50 [36] pretrained on ImageNet is used as the backbone architecture. For Amazon Reviews, the feature extractor is composed of three fully-connected layers each with 1000, 500, and 100 output units, and a single fully-connected layer with 100 input units and 2 output units is adopted for the label classifier. With Digits-Five, LeNet5 [32] and ResNet14 [36] without any adaptation are additionally investigated in two different manners: *Source Combined* and *Single Best*. In *Source Combined*, multiple source datasets are simply combined and fed into a model. In *Single Best*, we train the model with each source dataset independently and report the result of the best performing one. Likewise, ResNet50 and MLP consisting of 4 fully-connected layers with 1000, 500, 100, and 2 units are investigated without adaptation for Office-Caltech10 and Amazon Reviews, respectively.

#### Training details

We train our models for Digits-Five with Adam optimizer [37] with *β*_1_ = 0.9, *β*_2_ = 0.999, and the learning rate of 0.0004 for 100 epochs. All images are scaled to 32 × 32 and the mini-batch size is set to 128. We set the hyperparameters *α* = 0.01, and *K* = 1. For the experiments with Office-Caltech10, all the modules comprising our model are trained with the SGD-momentum optimizer with the weight decay of 0.001 and the momentum factor of 0.9. The learning rate for the feature extractors and the label classifiers are 0.0001 and 0.001, respectively. We scale all the images to 224 × 224 and set the mini-batch size to 48. All the other hyperparameters are kept the same as in the experiments with Digits-Five. For Amazon Reviews, we train the models for 50 epochs using Adam optimizer with *β*_1_ = 0.9, *β*_2_ = 0.999, and the learning rate of 0.0001. We set *α* = 0.1, *K* = 2, and the mini-batch size to 100.

### Performance evaluation

We evaluate the performance of Multi-EPL against the competitors. We repeat experiments for each setting five times and report the mean and the standard deviation. The results are summarized in Tables 3–5. In the tables, SC and SB indicate *Source Combined* and *Single Best*, respectively. Note that Multi-EPL provides the best accuracy in all the datasets, showing its superiority in both image datasets (Digits-Five and Office-Caltech10) and text datasets (Amazon Reviews). The enhancement is remarkable especially when MNIST-M is the target domain in Digits-Five, improving the accuracy by 13.20% compared to the state-of-the-art methods. It is also notable that Multi-EPL consistently achieves successful adaptation of multiple domains, while other state-of-the-art methods sometimes fail to adapt and even deteriorate the performance. The failure appears to be attributable to negative transfer [38], but we leave this issue as a future work.

**Table 3:** Classification accuracy on Digits-Five with and without domain adaptation.

Method	→T	→M	→S	→D	→U
LeNet5 (SC)	97.6±0.2	61.7±1.4	75.2±0.8	80.3±0.7	81.6±1.5
ResNet14 (SC)	98.2±0.3	63.5±0.8	79.1±1.6	92.9±0.5	94.5±0.3
LeNet5 (SB)	97.1±0.1	51.1±1.9	76.8±0.6	79.9±0.5	83.3±0.9
ResNet14 (SB)	97.1±1.0	49.5±1.3	81.4±0.7	91.8±0.5	91.5±2.7
DCTN	99.3±0.1	72.0±1.6	78.3±1.1	91.6±0.7	**98.4±0.2**
M^3^SDA	98.8±0.1	67.8±0.7	81.8±0.6	88.5±0.3	97.2±0.2
M^3^SDA-*β*	99.0±0.1	72.5±0.2	81.4±0.3	89.5±0.4	97.4±0.2
MDDA	98.8±0.4	78.6±0.6	79.3±0.8	89.7±0.7	93.9±0.5
LtC-MSDA	99.1±0.1	78.9±1.8	79.8±2.2	91.7±0.3	98.3±0.1
Multi-EPL	**99.4±0.1**	**92.1±0.2**	**85.4±0.3**	**95.1±0.1**	98.2±0.1

The letters T, M, S, D, and U stand for MNIST, MNIST-M, SVHN, SynthDigits, and USPS, respectively. For MDDA, we report the performances in [18, 19].

**Table 4:** Classification accuracy on Office-Caltech10 with and without domain adaptation.

Method	→A	→C	→D	→W
ResNet50 (SC)	95.47±0.25	91.59±0.51	99.36±0.78	99.26±0.37
ResNet50 (SB)	95.03±0.48	89.05±0.88	99.87±0.28	98.24±0.61
DCTN	95.05±0.24	90.60±0.71	**100.0±0.00**	99.46±0.62
M^3^SDA	95.14±0.31	93.59±0.40	99.49±0.53	99.86±0.19
M^3^SDA-*β*	94.36±0.26	91.70±0.71	99.75±0.35	99.39±0.15
LtC-MSDA	95.68±0.84	92.34±0.61	**100.0±0.00**	99.86±0.19
Multi-EPL	**96.23±0.13**	**93.52±0.49**	**100.0±0.00**	**99.93±0.16**

The letters A, C, D, and W stand for Amazon, Caltech, DSLR, and Webcam, respectively.

**Table 5:** Classification accuracy on Amazon Reviews with and without domain adaptation.

Method	→B	→D	→E	→K
MLP (SC)	79.76±0.70	82.18±0.59	84.42±0.27	87.23±0.51
MLP (SB)	79.00±0.92	80.38±0.61	84.76±0.45	87.46±0.36
DCTN	78.92±0.56	81.22±1.01	83.56±1.52	86.47±0.71
M^3^SDA	78.97±0.79	80.51±0.99	83.63±0.68	85.99±0.85
M^3^SDA-*β*	80.26±0.43	81.80±0.72	85.02±0.34	86.99±0.56
LtC-MSDA	76.73±0.79	78.03±1.92	79.51±2.09	81.49±2.38
Multi-EPL	**81.00±0.53**	**83.42±0.31**	**86.53±0.44**	**88.64±0.53**

The letters B, D, E, and K stand for Books, DVDs, Electronics, and Kitchen appliances, respectively.

**Fig 4:**
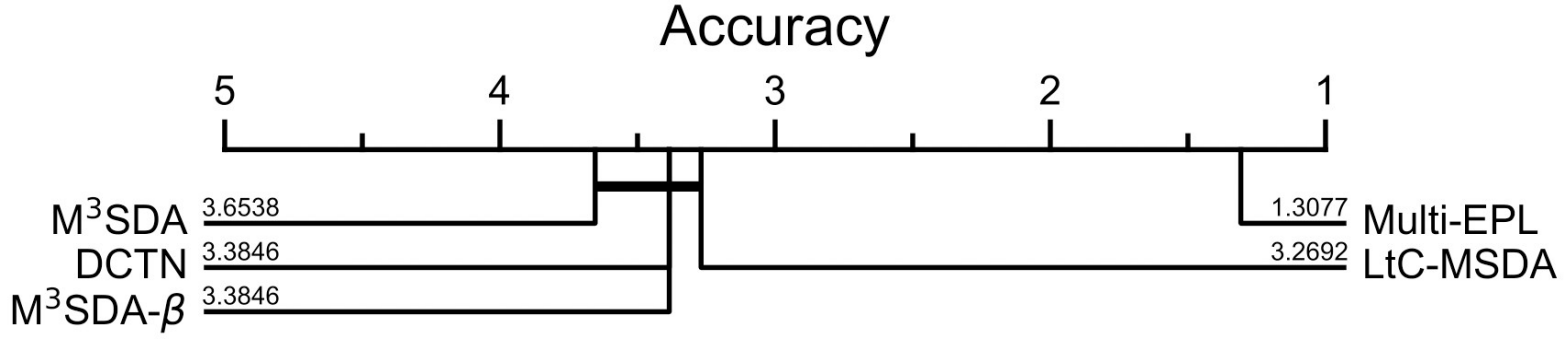
CD Diagram with various adaptation methods: DCTN, M^3^SDA, M^3^SDA-β, LtC-MSDA, and Multi-EPL.

We also illustrate the summary of the results in Fig 4 using CD (critical difference) diagram [39]. We tackled every single source and target scenario, and the five adaptation methods DCTN, M^3^SDA, M^3^SDA-*β*, LtC-MSDA, and Multi-EPL. It demonstrates that Multi-EPL gives significant performance enhancement compared to the existing methods.

### Ablation study

We perform an ablation study on Digits-Five to identify what exactly enhances the performance of Multi-EPL. We compare Multi-EPL with three of its variants: Multi-0, Multi-PL, and Multi-PL-ded. Multi-0 aligns moments regardless of the labels of the data. Multi-PL trains the model without ensemble learning. Multi-PL-Ded consists of four feature generators and four label classifiers, each of which is dedicated to each source domain.

The results are shown in Table 6. By comparing Multi-0 with Multi-PL, we observe that considering labels in moment matching plays a significant role in extracting domain-invariant features. The remarkable performance gap between Multi-PL and Multi-EPL verifies the effectiveness of ensemble learning. The overall accuracy of Multi-PL-Ded is much lower than that of Multi-PL or Multi-EPL; it demonstrates that the existing methods that assign individual networks for each source domain deteriorate not only the performance but also the model efficiency.

**Table 6:** Experiments with Multi-EPL and its variants.

Method	→T	→M	→S	→D	→U
Multi-0	98.8±0.1	67.8±0.7	81.8±0.6	88.5±0.3	97.2±0.2
Multi-PL	99.3±0.1	90.2±0.5	83.7±0.4	94.4±0.2	98.0±0.2
Multi-PL-Ded	**99.4±0.1**	65.5±3.8	29.4±0.7	41.0±1.3	**98.6±0.2**
Multi-EPL (n = 2)	**99.4±0.1**	**92.0±0.2**	85.4±0.3	95.1±0.1	98.2±0.1
Multi-EPL (n = 3)	99.3±0.1	91.6±0.9	85.9±0.7	**95.2±0.2**	98.5±0.1
Multi-EPL (n = 4)	99.3±0.1	91.5±1.2	**86.9±0.9**	95.1±0.1	98.5±0.1

### Effects of ensemble

We evaluate the performance on Digits-Five while varying the number *n* of pairs of feature extractor and label classifier. The results are summarized in Table 6. While an ensemble of two pairs gives much better performance than the model with a single pair, using more than two pairs does not bring remarkable improvement, except for the case of SVHN being the target dataset. We presume that the overfitting due to the excessive number of parameters has hindered the further improvement. We leave the task of figuring out proper regularization methods for the ensembles as a future work.

### Parameter efficiency

**Fig 5:**
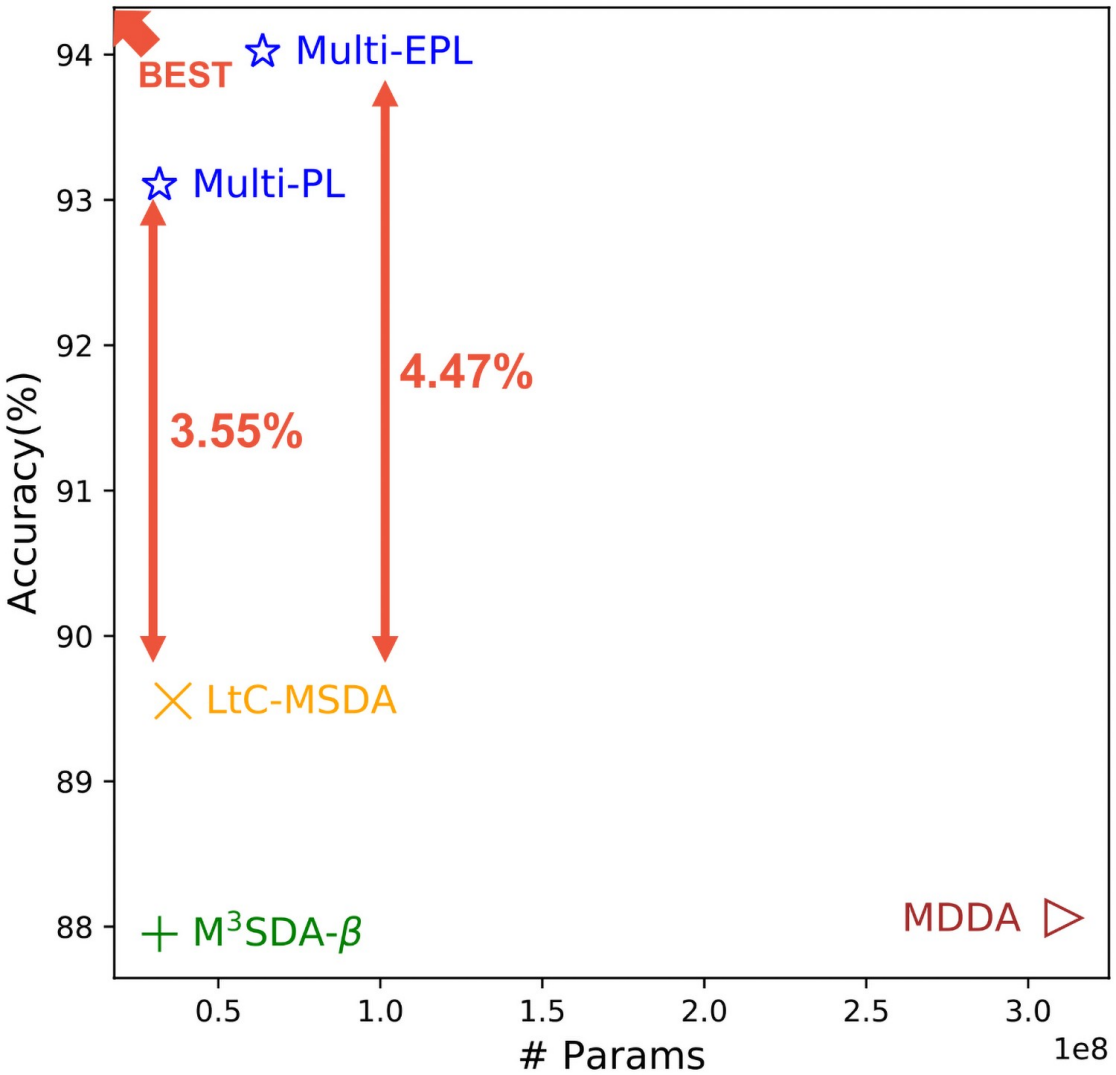
The number of parameters and the model accuracy of the MSDA methods.

We compare the number of parameters and performance of Multi-EPL with other state-of-the-art methods to demonstrate Multi-EPL’s efficient usage of the model complexity. Fig 5 illustrates the number of model parameters and the average accuracy of each method that are evaluated with the Digits-Five dataset. Multi-PL is the variation of Multi-EPL that does not exploit the ensemble technique. Comparing Multi-PL and LtC-MSDA, the superiority of the proposed method is proved under the fair model complexity. On the other hand, the significant performance enhancement that the ensemble learning technique has made in Multi-EPL demonstrates that Multi-EPL greatly benefits from the additional model parameters, while MDDA has made little performance improvement even though it requires much more model parameters.

### Visualization

**Fig 6:**
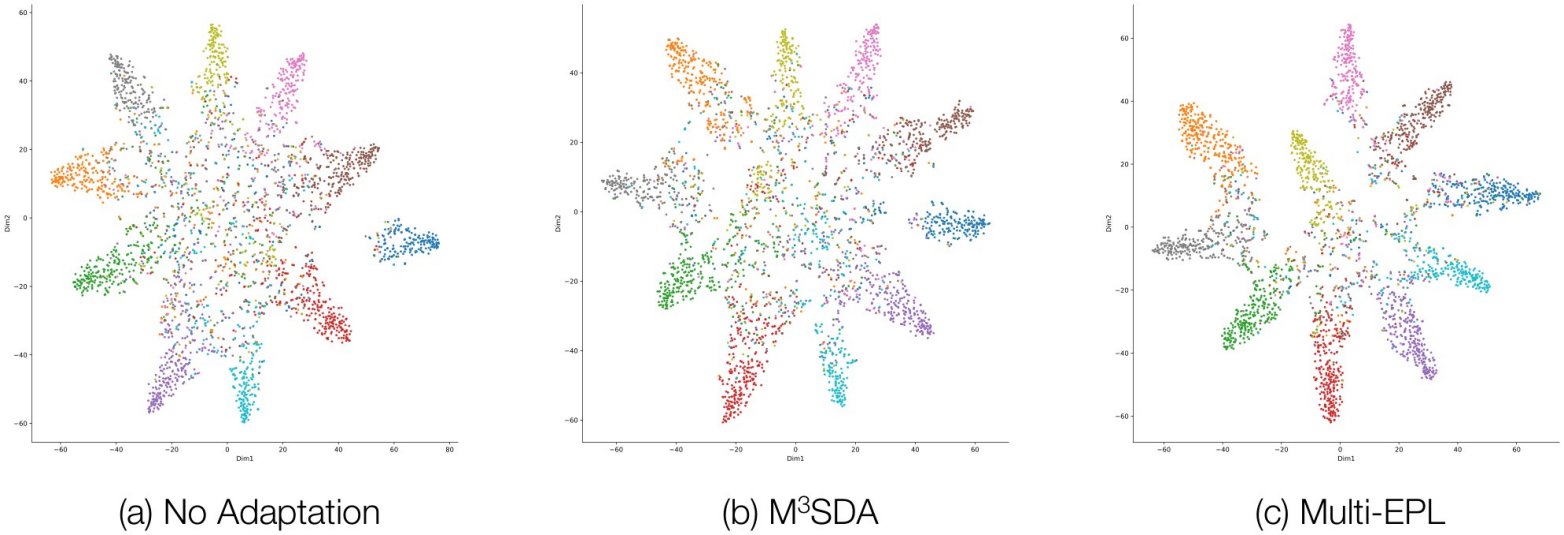
T-SNE visualization of the features from different adaptation methods.

We visualize the features from distinct adaptation methods using T-SNE [40] to verify the effect of label-wise moment matching. Fig 6 shows the feature distributions when no adaptation method, M^3^SDA, and Multi-EPL are applied, respectively. All the experiments are conducted with Digits-Five with MNIST-M as the target dataset. Each color in Fig 6 stands for a label.

Note that Multi-EPL clearly separates features with different labels, while other do not; this explains the outstanding performance of Multi-EPL.

## Conclusion

We propose Multi-EPL, a novel framework for the multi-source domain adaptation problem. Multi-EPL overcomes the problems in existing methods of not directly addressing conditional distributions of data *p*(**x**|*y*), not fully exploiting the knowledge of target data, and having redundancy in model networks. Multi-EPL aligns data from multiple source domains and the target domain considering the data labels, and exploits pseudolabels for unlabeled target data. Multi-EPL further enhances the performance by generating an ensemble of multiple feature extractors. Our framework exhibits superior performance on both image and text classification tasks. Considering labels in moment matching and adding ensemble learning are shown to bring remarkable performance enhancement through ablation study. Future works include extending our approach to other tasks such as regression, which may require modification in the pseudolabeling method.
